# Crashworthiness Optimization Design of Aluminum Alloy Thin-Walled Triangle Column Based on Bioinspired Strategy

**DOI:** 10.3390/ma13030666

**Published:** 2020-02-02

**Authors:** Kangjie Li, Yixiong Feng, Yicong Gao, Hao Zheng, Hao Qiu

**Affiliations:** 1 State Key Laboratory of Fluid Power and Mechatronic Systems, Zhejiang University, Hangzhou 310027, China; 21825116@zju.edu.cn (K.L.); fyxtv@zju.edu.cn (Y.F.); 11725066@zju.edu.cn (H.Q.); 2 Key Laboratory of Advanced Manufacturing Technology of Zhejiang Province, Zhejiang University, Hangzhou 310027, China; 3 Hangzhou Innovation Institute, Beihang University, Hangzhou 310052, China; Haozheng@zju.edu.cn

**Keywords:** crashworthiness, fractal design, thin-walled column, energy absorption

## Abstract

Aluminum alloy thin-walled structures have been well used in applications of energy absorption. In the present work, a bioinspired design strategy for aluminum alloy thin-walled structures is proposed to improve the performance of out-of-plane crashworthiness by altering the material distribution. According to the proposed strategy, a novel fractal thin-walled triangle column (FTTC) is designed, which is composed by iteratively applying the affine transformation of a base triangle up to 2nd-order. The finite element model is established to investigate the out-of-plane crashworthiness of FTTC and validated by experiment results. The numerical analysis of the crashworthiness of FTTC with different fractal orders (0th, 1st and 2nd) are performed, and the results show that 1st- and 2nd-order FTTC enhance the energy absorption of structures and crush force efficiency. In particular, 2nd-order FTTC has better energy absorption ability due to the optimal distribution of materials, which are efficiently organized by the proposed bioinspired design strategy. In addition, a parameter study is performed to investigate the effect of FTTC geometric details on the crushing procedure. The collapse mode shows that it tends to change from unstable to stable with the increase in thickness and side length and the decrease in height. Moreover, a positive relevant relationship is identified between the thickness and the crashworthiness for FTTC.

## 1. Introduction

Aluminum alloy thin-walled structures, due to their high energy absorption efficiency and lightweight structures, are an essential requirement in many industries. Aerospace, elevator, automotive, offshore structures, and liquid storage tanks inevitably use energy absorbers [1,2,3,4,5,6]. Different section shapes of aluminum alloy thin-walled structures such as square and hexagonal have been well documented [7,8], and the majority of research focuses on the even sides. However, odd sides’ tubes such as triangular are also widely used in bridges and buildings et al. Therefore, more attention has been paid to study these structures in recent years. Alexander [9] presented the first theoretical model of the thin-walled structures’ plastic collapse, which modeled the procedure as one stationary hinge and folding wave. Abramowicz [10] expanded the theory by proposing the effective crushing distance concept. Wierzbicki [11,12] proposed the Super Folding Element (SFE) theory, which combined the concepts from plasticity with moving hinge line collapse theories. Chen [13] proposed a simplified SFE based on energy mechanisms. Zhang [14] studied the collapse performance of X-shaped elements with various angles and modified the inextensional mode of corner element. Later, he established the theoretical collapse model of a three-plate element with an arbitrary central angle [15]. With the advances of the thin-walled structure theories and the maturity of the computer simulation, the thin-walled structures of varies cross-section were extensively studied. Wang [16] inferred the theoretical prediction of the mean crushing force of the thin-walled multi-cell structures based on the simplified super folding element theory. Then he investigated a combined five-cell thin-walled structure assembled in high speed. Tran [17] proposed a nested tubular thin-walled structure, and studied the crush behavior and energy absorption performance under dynamic axial loading. Zhang [18] proposed a novel type of self-locking multi-cell structure. On the other hand, the dynamic and static experimental tests were conducted to look into the energy absorption characteristics. Gui [19] presented a comprehensive crashworthiness design method of the automotive structure plastic frame model, which produced a thin-walled beam with an arbitrary cross-sectional shape. Fang [20] proposed a topology optimization method based on a modified artificial bee colony algorithm in order to make more efficient use of the material of the multi-cell tube under out-of-plane crushing. Zhang [21] designed a quadric-arc multi-cell honeycomb and investigated the in-plane energy absorption property and dynamic crushing behavior under various impact loads.

Recently, there has been a trend to adopt bioinspired design principles in the structure design. Nature employs fractal designs to serve a mechanical function in many cases [22,23,24]. Fractal geometry is one of the most suitable choices to improve the collapse performance of the thin-walled structure. Inspired by the ’pomelo peel’s unique microstructure, Zhang [25] constructed a new hierarchical honeycomb. Xu [26] proposed a new self-similar hierarchical column and the crashworthiness was studied to improve the performance. Inspired by Koch topology, Wang [27] presented a specific multi-corner fractal structure with the highest specific energy absorption performance. Zhang [28] proposed a bio-inspired hierarchical circular tube to enhance the structural crashworthiness performance. Later, Xu [29] analyzed and optimized the hybrid multi-cell structures. Dadrasi [30] presented steel thin-walled square columns, which were reinforced with three types of reinforcers. Zhang [31] presented a fractal-appearing self-similar regular hexagonal hierarchical honeycombs, and achieved further improvement by design of the fractal geometries. Ajdari [32] investigated the mechanical behavior of two-dimensional hierarchical honeycomb structures by using analytical, numerical and experimental methods.

As mentioned above, the existing studies mainly focused on the application scope and summarizing it into a design method remains unexplored. In the present work, a bioinspired design strategy of the cross-section of the thin-walled column is proposed. A novel type of triangle thin-walled columns is presented based on the strategy. The out-of-plane crashworthiness theoretical model is established. The finite element analysis is carried on to investigate the mechanical characteristics of various structures. In addition, parameter studied is done to find the effect of different design variables, such as columns height, side length, and panel thickness. There are three main contributions of this paper. Firstly, a bioinspired design strategy is proposed, which provides new insight into the designing of thin-walled columns with high energy absorption performance. Secondly, a novel fractal thin-walled triangle column up to 2nd-order is presented. Thirdly, the energy absorption properties in theoretical and numerical manners are investigated, and a positive relevant relationship is identified between the thickness and the crashworthiness.

The rest of the paper is organized as follows. Section 2 gives the numerical model of the structure and conduct an experimental validation. The theoretical models of the columns are established in Section 3. The comparison of theoretical and simulation results of different orders is discussed in Section 4. Section 5 conducts the parametric studies of crashworthiness to identify the effects of side length, height and wall thickness.

## 2. Numerical Models and Validation

### 2.1. Process Overview

**Figure 1:**
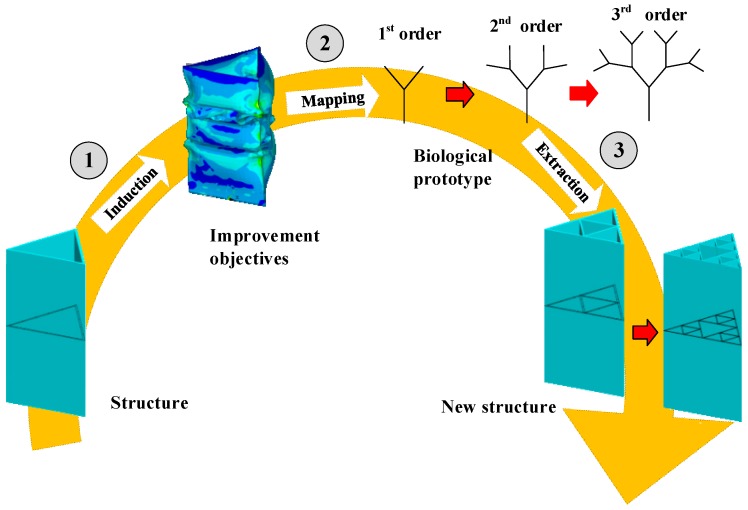
Main steps of the top-down bioinspired design method.

The goal of this study is to develop a bioinspired design method to improve the crashworthiness performance of aluminum alloy thin-walled structures. Based on Ref [33], a top-down bionic process is developed, which starts from technical problems and finds solutions through natural systems. The main steps of the proposed method are shown in Figure 1. For a given profile of a thin-walled column, the design method is organized in 3 steps. Firstly, the insufficiencies of the existing method are found and refined into some improvement objectives. Secondly, the promotion goal is mapped into a real biological prototype. Finally, by studying how biology copes with this problem, a corresponding design strategy is extracted to guide the fabrication.

### 2.2. Bioinspired Strategy

**Figure 2:**
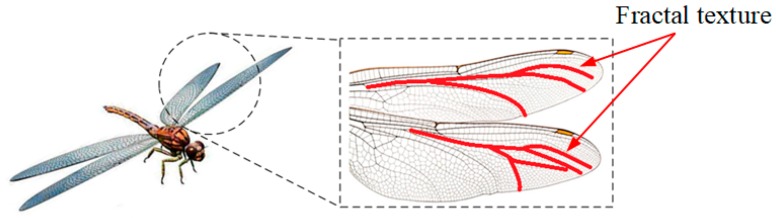
The fractal texture of the wings of dragonflies.

Fracture structures have emerging applications in strength requirement conditions, which are inspired by natural systems such as the wings of dragonflies, as shown in Figure 2. The definition of a fractal is a shape that repeats itself in a different range of scales. A fractal is a subset of an n-dimensional metric space that is approximately or perfectly self-similar, whose Hausdorff dimension exceeds its topological dimension. Based on the contraction mapping theory [34], the fractal set as an attractor Δ can be obtained from a set of affine transformations *F*. The attractor Δ is a union set that is scaled copies of the initial set, which can be expressed as follows:

(1) $$\left\{ \begin{array}{l} \Delta =\overset{\infty }{\underset{n=1}{\cup }}\Delta_{n}\left(F,\Delta_{n-1}\right)\\ F=\left(f_{0},f_{1},\cdots ,f_{m}\right) \end{array}\right.$$

where *n* is the number iterations, *f* is the subset of transformation, *m* is the number of transformations.

Based on contraction mapping theory, the bio-inspired design strategy is illustrated as follows:

Step 1. Define the affine transformation matrix F.

The two-dimensional affine transformation *f_i_* in Euclidean XY plane is defined as:

(2) $$f_{i}=\lambda_{i}\mu_{i}r_{i}x_{i}+\delta_{i}=\lambda_{i}\left[\begin{array}{cc} \mu_{1i} & 0\\ 0 & \mu_{2i} \end{array}\right]\left[\begin{array}{cc} \cos\theta_{i} & -\sin\theta_{i}\\ \sin\theta_{i} & \cos\theta_{i} \end{array}\right]\left[\begin{array}{c} x_{i}\\ y_{i} \end{array}\right]+\left[\begin{array}{c} \delta_{xi}\\ \delta_{yi} \end{array}\right],i=0,\cdots ,m$$

where $\mu_{1i}$ and $\mu_{2i}$ are reflection control coefficient, respectively. $\theta_{i}$ is the rotation angle, $\delta_{xi}$ and $\delta_{yi}$ are the displacement along the x-axis and y-axis, respectively.

Step 2. Perform the Iteration According to the Affine Transformation Matrix F.

After the *n*th iteration (*n* initial to be 0) of Step 2, the new pattern based on the previous one is got. The pattern vertices are donated by vector *p*. According to the practical needs, the hierarchical transformation *L* is applied, which connects the vertices of the present and the previous one.

(3) $$L\left(p_{n},p_{n-1}\right)$$

Step 3. Set Operation.

Select whether to retain the intersection part (if any). If it is kept, Equation (5) is employed to fulfill the procedure. Otherwise, use Eqaution (6).

(4) $$\Delta_{n}=\overset{m}{\underset{i=0}{\cup }}f_{i}\left(\Delta_{n-1}\right)$$

(5) $$\Delta_{n}=∁\left(\overset{m}{\underset{i=0}{\cap }}f_{i}\left(\Delta_{n-1}\right)\right)$$

In case the graph after Step 3 does not satisfy the requirement, go Step 1 and come again. If the expected iteration steps reached, finish. Otherwise, *n* = *n* + 1, go Step 2 and perform the next iteration.

**Figure 3:**
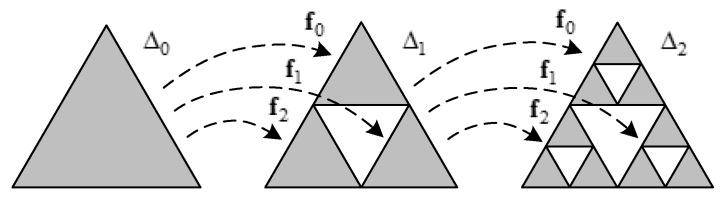
The example of acquiring the designed shape through two iterations of the procedure.

Figure 3 shows an example of acquiring the designed shape through two iterations of the above procedure.

### 2.3. Geometry Model

According to the bioinspired fractal design strategy, the fractal pattern of dragonfly wings is employed. Based on the odd side’s regular triangle, a series of fractal sections with different orders are created as shown in Table 1. They are named FTTC series, which is an abbreviation of fractal thin-walled triangle column. The initial triangle column is expressed as the 0th-order structure. D is the side length of the smallest triangle. Q is the total side number of all triangles. L is the total length of the sides. t is the thickness. More configuration details are shown in Table 1.

### 2.4. FE Models and Validation

To investigate the crashworthiness of FTTC structure, total energy absorption, specific energy absorption, and crush force efficiency are employed [35,36].

Total energy absorption can be obtained by the integral of the load-displacement curve. For a given displacement *d*, it can be calculated as:

(6) $$EA\left(d\right)=\int_{0}^{d}F\left(x\right)dx$$

where *F(x)* is the crushing force at crushing distance *x*.

Specific energy absorption is the per unit mass energy absorbed of a specimen. It is the main indicator to distinguish the energy absorption efficiency of different materials. It is formulated as:

(7) $$SEA=\frac{EA}{m}$$

where *m* is the total mass of the structure. *EA* is the value of energy absorption.

The mean crush force that can be obtained as:

(8) $$MCF=\frac{EA}{d}$$

Thus, the crush force efficiency definition is calculated as:

(9) $$CFE=\frac{MCF}{PCF}\times 100\%$$

where *PCF* is the peak force during collapse procedure. *CFE* is an indicator that reflects the load uniformity of a structure.

**Figure 4:**
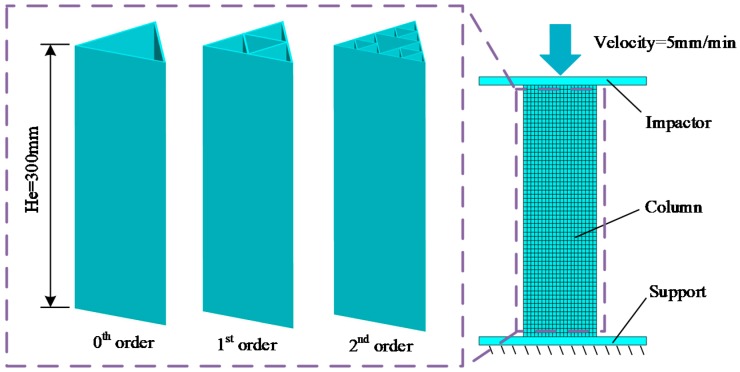
The FE model of the novel fractal structures.

The FE model of the novel fractal structures of FTTC in the out-of-plane crush is shown in Figure 4. The 0th fractal column is picked to do the FE model validation. The height *H_e_* and the side length *D*_0_ of the 0th fractal column are 300 mm and 100 mm, respectively. The thickness *t*_0_ is set to 2 mm. The bottom of the column is supported by a rigid body. A rigid impactor is moving to the top end of the column with a constant speed of 5 mm/min. The total collapse distance is 150 mm.

**Figure 5:**
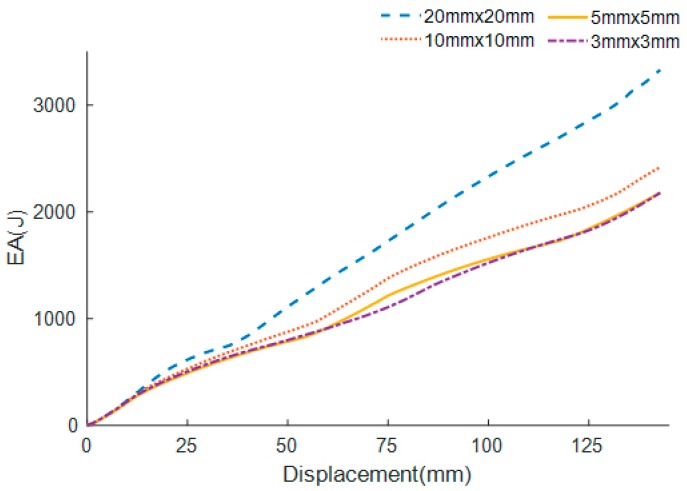
The SEA convergence behaviors of the 0th-order FTTC with various element sizes.

To simulate the collapse procedure of FTTC series, the nonlinear explicit FE code LS-DYNA is employed. All FE models are based on Belytschko-Tsay four-node shell elements. It has five integration points across the thickness and one integration point in the plane [35]. To acquire a fair balance between the numerical analysis computational cost and the accuracy, convergence analyses are carried out. For the buckling of the specimen under axial loading, an automatic single-surface contact is used. The automatic surface-to-surface contact is picked to simulate the contact between the rigid wall and the specimen. The friction coefficients of the dynamic and static are set as 0.2 and 0.3, respectively [27]. The material of specimens is aluminum alloy A6061-O with properties [37] listed in Table 2. The specimen is represented by a piecewise linear plastic strain hardening material (MAT_24). The impactor and rigid wall are modeled by rigid material (Mat_20). Additionally, due to the weak strain sensitivity of the material, the effect of strain rate is neglected [32]. The hourglass energy of the columns is checked on account of the element high distortion [38]. It is found that it is less than 2% of the internal energy, which means the correctness of the analysis is ensured. Figure 5 shows the SEA convergence behaviors of the 0th-order FTTC with various element size. It can be observed that the SEA gradually converges when the element dimension decreases to 5.0 mm × 5.0 mm. Therefore, this 5.0 mm × 5.0 mm element size is selected.

**Figure 6:**
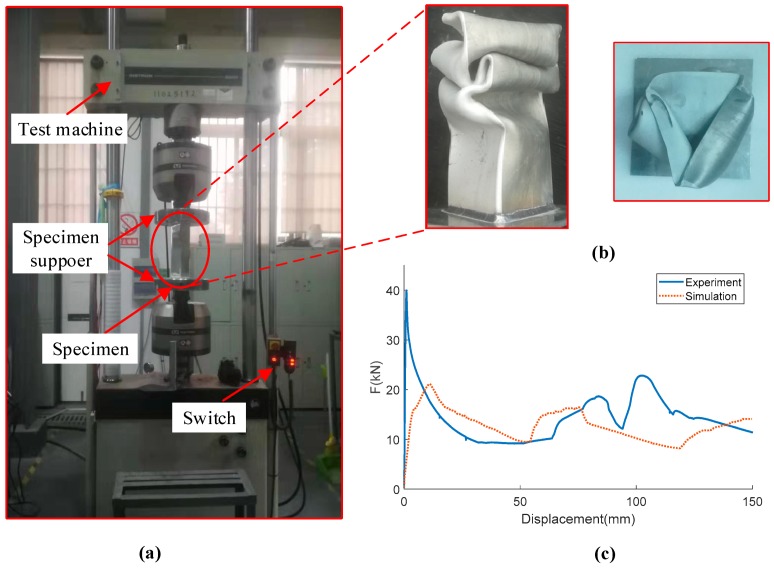
(**a**) Photograph of the experiment set-up, (**b**) deformed shapes of the specimen, and (**c**) comparison of force-displacement.

To verify the accuracy of the FE model, the experiment is performed. The 0th-order column is fabricated by cutting and welding. The quasi-static compression test is performed using WDW-100 compression testing general machine as shown in Figure 6a. The specimen is placed between two flat plates, and 5 mm/min stroke is used in the test. The collapse displacement is 150 mm. The displacement and crushing force data are recorded by the sensor. The deformed shape and the force-displacement curves of simulation and experiment are illustrated in Figure 6b,c. All the forces experience a sharp rise in the beginning, followed by a fluctuation during the collapse period. Even though the experimental curve is not strictly consistent with the numerical curve, it still captures the general trend. Moreover, the comparison of energy absorption indicators is shown in Table 3. The experimental test indicators are consistent with numerical with the maximum relative error of 12.7%. Therefore, the FE model of the FTTC structure is reliable in predicting crushing behavior.

## 3. Theoretical Model Establishment

Based on the Super Folding Element theory [12], the external work done during the deformation is equal to the energy dissipation. The dissipation of energy consists of bending energy and membrane energy. It can be expressed as:

(10) $$P_{m}\times 2H\times \eta =E_{bending}+E_{membrane}$$

where *P_m_* denotes the average crush force, *H* is the half fold length, *η* represents the effective crushing distance coefficient. *E_bending_* and *E_membrane_* are the bending dissipation energy and the membrane dissipation energy respectively.

In the experiment, the mean crush force is calculated by:

(11) $$P_{e}=\frac{1}{S_{d}}\int_{0}^{S_{d}}P(x)dx$$

where *P_e_* is the experimental mean crush force, *S_d_* is the effective crushing length, *P(x)* is the crushing force at the distance of *x*.

The bending energy is evaluated by the energy dissipation in the plastic hinge lines [13]. Due to theoretical deduction, the panel should be perfect flatten during the collapse. The bending energy for a multi-cell column is obtained as:

(12) $$E_{bending}=2\pi M_{0}\sum_{i=1}^{p}b_{i}$$

where *M*_0_ = *σ*_0_*t*^2^/*4* represents the wholly plastic bending moment per unit width, *b* is the panel width, *p* is the number of the panel in the multi-cell column. *σ*_0_ refers to the flow stress of the material, and *t* is the thickness of the column.

(13) $$\sigma_{0}=\sqrt{\frac{\sigma_{y}\sigma_{u}}{1+n}}$$

where *σ_y_*, *σ_u_*, are the yield stress of the material and the ultimate stress of the material respectively. *n* is power law exponent.

For the 0th FTTC, it has three panels width *D*_0_ and the bending energy is calculated as:

(14) $$E_{bending}^{0th}=\frac{3}{2}\pi \sigma_{0}D_{0}t_{0}{}^{2}$$

For the 1st FTTC, it has nine panels width *D*_1_ and the bending energy is calculated as:

(15) $$E_{bending}^{1st}=\frac{9}{2}\pi \sigma_{0}D_{1}t_{1}{}^{2}$$

For the 2nd FTTC, it has 27 panels width *D*_2_ and the bending energy is calculated as:

(16) $$E_{bending}^{2nd}=\frac{27}{2}\pi \sigma_{0}D_{2}t_{2}{}^{2}$$

**Figure 7:**
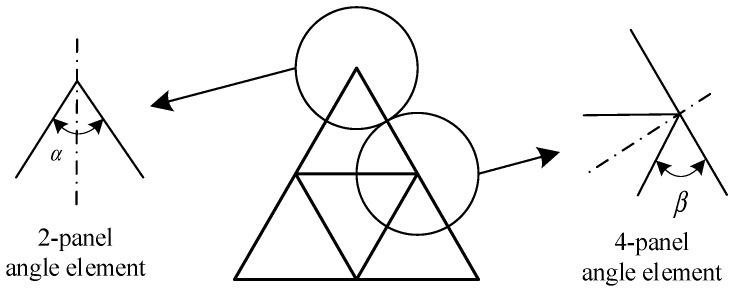
Cross-section of FTTC and two typical angle elements.

According to Ref [12,39], the irregularity section is divided into a small element and the fractal columns’ cross-section consists of two-panel and four-panel angle element, as shown in Figure 7. The membrane energy can be calculated as:

(17) $$E_{membrane}^{2-panel}=4M_{0}\frac{H^{2}}{t}\cos(\frac{90^{{}^{\circ}}-\alpha }{2})$$

(18) $$E_{membrane}^{4-panel}=8M_{0}\frac{H^{2}}{t}\left(1+\frac{1}{\cos\beta }\right)$$

where *α* and *β* are the angles in the element.

There are three two-panel angle elements in the 0th profile of the column. Therefore, for the 0th-order FTTC, the membrane energy is calculated as:

(19) $$E_{membrane}^{0th}=3\sigma_{0}t_{0}H_{0}{}^{2}\cos\frac{90^{{}^{\circ}}-\alpha }{2}$$

For the 1st-order FTTC, there are three two-panel angle elements and three four-panel angle elements. As a result, the membrane energy is calculated as:

(20) $$E_{membrane}^{1st}=3\sigma_{0}t_{1}H_{1}{}^{2}\left(\cos\frac{90^{{}^{\circ}}-\alpha }{2}+2+\frac{2}{\cos\beta }\right)$$

The profile of the 2nd-order FTTC constitutes of three two-panel elements and twelve four-panel elements. The membrane energy is calculated as:

(21) $$E_{membrane}^{2nd}=3\sigma_{0}t_{2}H_{2}{}^{2}\left(\cos\frac{90^{{}^{\circ}}-\alpha }{2}+8+\frac{8}{\cos\beta }\right)$$

The calculation of theoretical solution of the average crushing force is introduced in this section, taking the 0th fractal column as an example. Substituting Equation (15) and Equation (20) into Equation (11). The mean crush force can be obtained as:

(22) $$P_{m}^{0th}=\frac{3}{4H_{0}\eta }\pi \sigma_{0}D_{0}t_{0}{}^{2}+\frac{3}{2\eta }\sigma_{0}t_{0}H_{0}\cos\frac{90^{o}-\alpha }{2}$$

The half folding wavelength is determined by the stationary condition of the mean crushing force:

(23) $$\frac{\partial P_{m}^{0th}}{\partial H_{0}}=0$$

Therefore

(24) $$H_{0}=\sqrt{\frac{\pi D_{0}t_{0}}{2\cos\frac{90^{{}^{\circ}}-\alpha }{2}}}$$

Substituting Equation (25) into Equation (23), the average crush force in quasi-static loading can be calculated as:

(25) $$P_{m}^{0th}=\frac{3\sigma_{0}t_{0}}{4\eta }\sqrt{2\pi D_{0}t_{0}\cos\frac{90^{{}^{\circ}}-\alpha }{2}}+\frac{3\sqrt{2}\sigma_{0}t_{0}}{4\eta }\sqrt{\pi D_{0}t_{0}\cos\frac{90^{{}^{\circ}}-\alpha }{2}}$$

Using the same procedure, the mean crush force of the other fractal columns can be expressed as:

(26) $$P_{m}^{1st}=\frac{9\sigma_{0}t_{1}}{4\eta }\sqrt{\frac{2\pi D_{1}t_{1}}{3}\left(\cos\frac{90^{{}^{\circ}}-\alpha }{2}+2+\frac{2}{\cos\beta }\right)}+\frac{3\sigma_{0}t_{1}}{2\eta }\sqrt{\frac{3\pi D_{1}t_{1}}{2}\left(\cos\frac{90^{{}^{\circ}}-\alpha }{2}+2+\frac{2}{\cos\beta }\right)}$$

(27) $$P_{m}^{2nd}=\frac{9\sigma_{0}t_{2}}{4\eta }\sqrt{2\pi D_{2}t_{2}\left(\cos\frac{90^{{}^{\circ}}-\alpha }{2}+8+\frac{8}{\cos\beta }\right)}+\frac{9\sigma_{0}t_{2}}{2\eta }\sqrt{\frac{\pi D_{2}t_{2}}{2}\left(\cos\frac{90^{{}^{\circ}}-\alpha }{2}+8+\frac{8}{\cos\beta }\right)}$$

## 4. Crashworthiness Comparison

For the sake of maintaining the same material usage, all the specimens have the same mass (same section area). According to Table 1, the dimensions of the FTTC series are listed in Table 4.

The comparison of the FEA and theoretical prediction of the average crush force is illustrated in Table 5. It can be seen the maximum relative error of theoretical to the simulation is 14.54%. For the progressive folding 0th-order one, the error is relatively low. Therefore, the theoretical model is sufficient accuracy to predict PCF.

**Figure 8:**
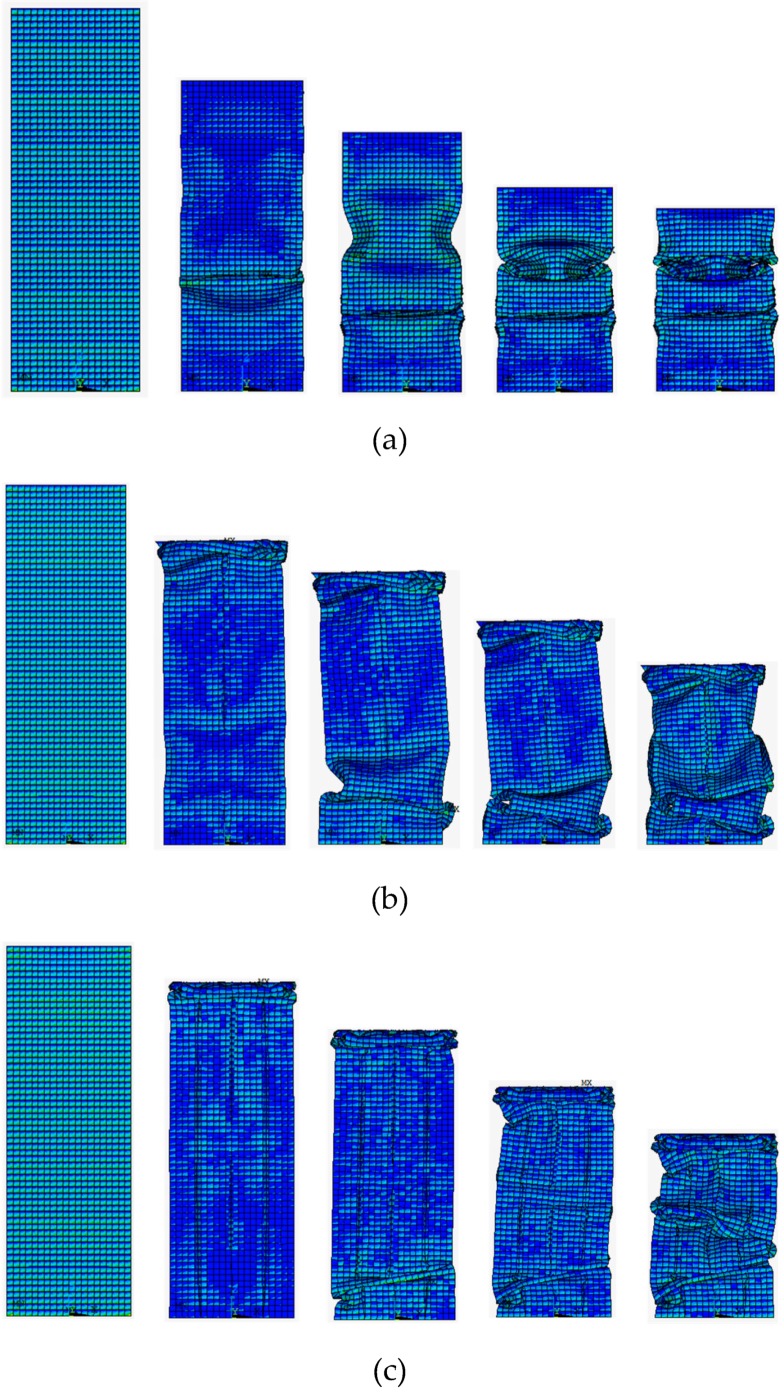
Deformation process of three FTTC columns: (**a**) 0th-order; (**b**) 1st-order; (**c**) 2nd-order.

**Figure 9:**
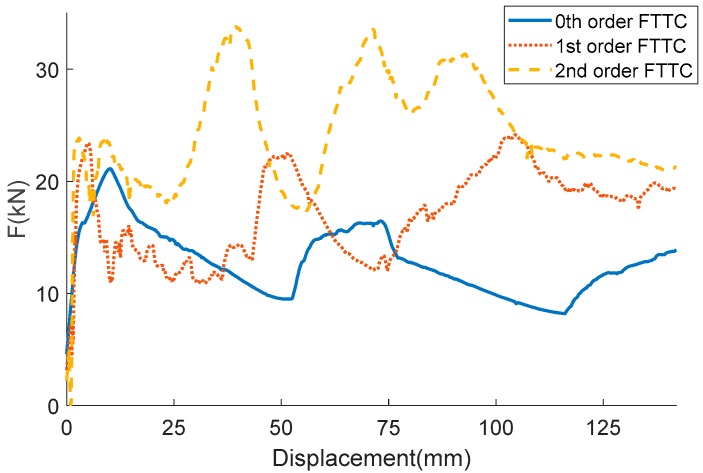
Force–displacement curves of different FTTC columns.

The deformation process of the three columns is illustrated in Figure 8. As well as the corresponding force-displacement curves of the three columns are illustrated in Figure 9. It can be observed that columns have an acute peak at the beginning stage, then followed by a fluctuating load, which is the most energy dissipated place. The 0th-order column exhibits a typical progressive folding. The column shows a bending tendency with the increase of the fractal order. Thus, the force-displacement behave more irregular. The comparison of the indicators of the crashworthiness is shown in Table 6. Energy absorption is a key indicator that reflects energy absorption performance and SEA is employed to evaluate the energy absorption performance. As shown in Table 6, the 0th-order FTTC has the smallest SEA of 3.76 kJ/kg, and the 2nd-order FTTC has the biggest SEA of 7.13 kJ/kg, which is 89.6% higher than the 0th-order. There is an average increase of 30%-40% SEA via increasing the fractal order. CFE represents the load uniformity of a structure. The 2nd-order FTTC shows the most uniformity load due to its high rigidity. It can be observed that the CFE becomes larger with the increase of the fractal order.

## 5. Parametric Study of Crashworthiness

### 5.1. Collapse Modes

**Figure 10:**
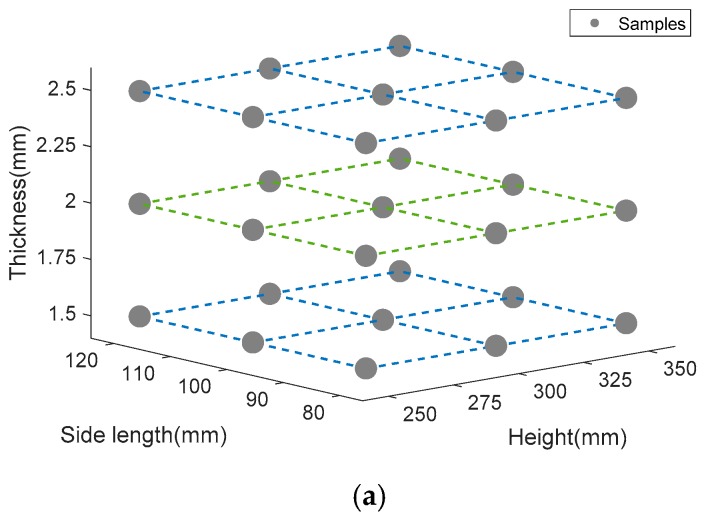

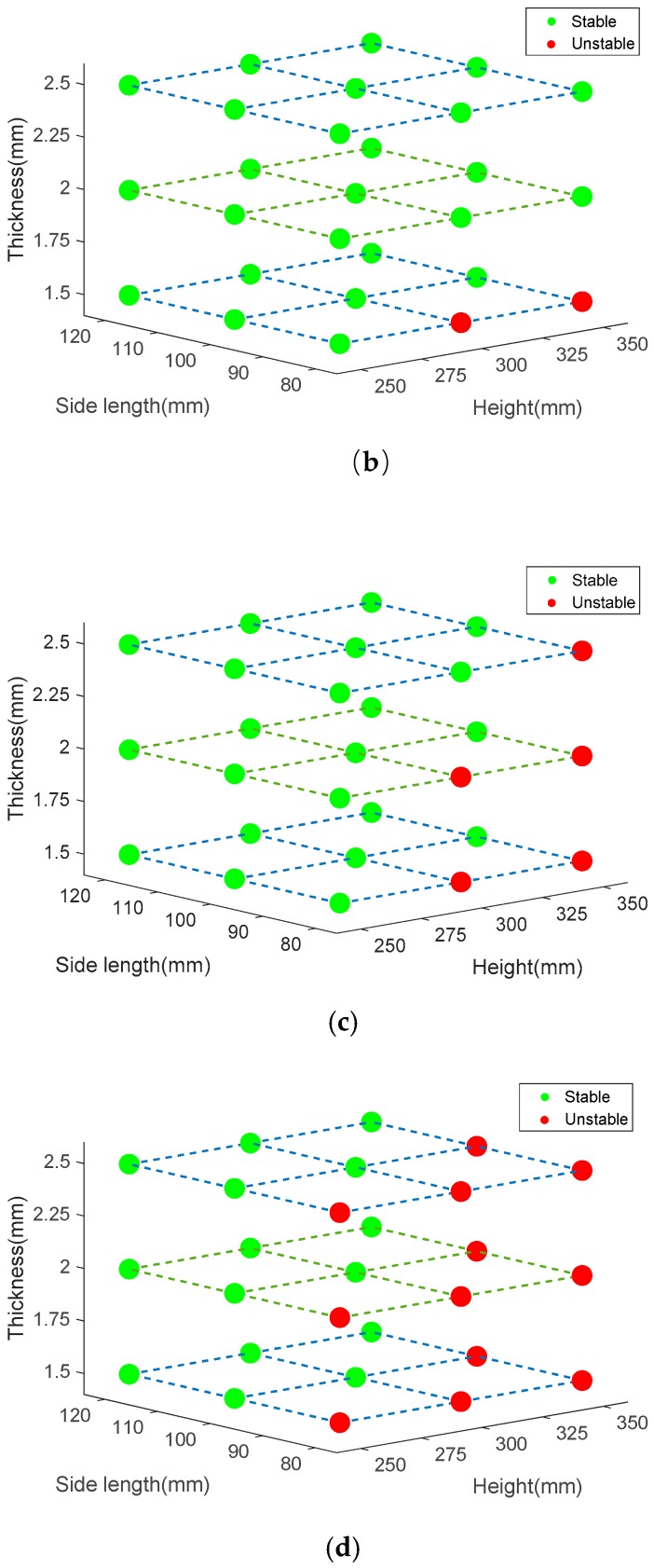
(**a**) Sampling points of the three parameters, (**b**) collapse mode diagram of 0th-order column, (**c**) collapse mode diagram of 1st-order column, (**d**) collapse mode diagram of 2nd-order column.

According to Ref [27,40,41], there are two different collapse modes during bucking. Stable mode is the expected form of progressive top-down bucking. Unstable mode represents the local or global bending during the bucking. There are three main design variables that affect the collapse mode, initial side length, column height, and thickness. It is necessary to investigate the relationship between collapse mode and design variables. Initial side length *D*_0_ takes the value of 80 mm, 100 mm, and 120 mm. Column height *H_e_* takes the value of 250 mm, 300 mm, and 350 mm. Thickness *t*_0_ takes the value of 1.5 mm, 2 mm, 2.5 mm. The relationship of between design variables and the collapse mode is illustrated in Figure 10. Figure 10a shows the sampling points of the three parameters. Figure 10b shows the collapse mode diagram of 0th-order column. Figure 10c shows collapse mode diagram of 1st-order column. Figure 10d shows collapse mode diagram of the 2nd-order column. It can be found that the fractal order has a significant influence on the collapse mode of the FTTC. With the increase of fractal order, FTTC has an unstable tendency. Moreover, increasing the thickness and side length contributes to a stable collapse.

### 5.2. Effect of Thickness

**Figure 11:**
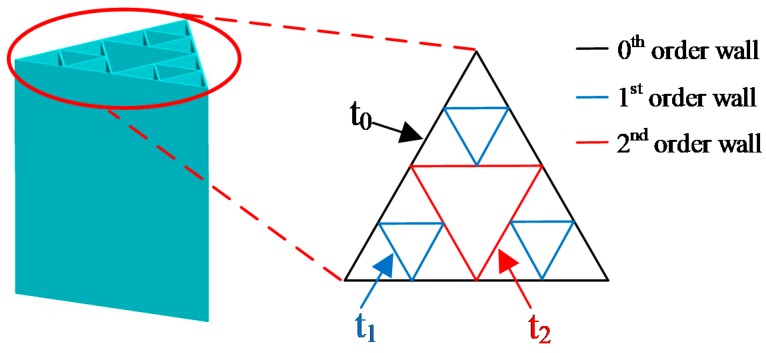
Illustration of the thickness of different level walls.

In the 2nd-order fractal column, changing the panel thickness of the 1st- and 2nd-order provides an additional way in managing the material distribution. The coefficient that reflects the effect of wall thickness change between fractal orders is defined as:

(28) $$\{\begin{array}{c} \varphi_{1}=\frac{t_{1}}{t_{0}}\\ \varphi_{2}=\frac{t_{2}}{t_{0}} \end{array}$$

where *t*_0_ is the 0th-order wall thickness, *t*_1_ is the 1st-order wall thickness, and *t*_2_ is the 2nd-order wall thickness, as shown in Figure 11. *t*_0_ is set 0.889 mm as the initial value, and the factor design of five levels (0.756 mm, 0.822 mm, 0.889 mm, 0.956 mm, 1.022 mm) is utilized to sample the design space of *t*_1_, and *t*_2_.

**Figure 12:**
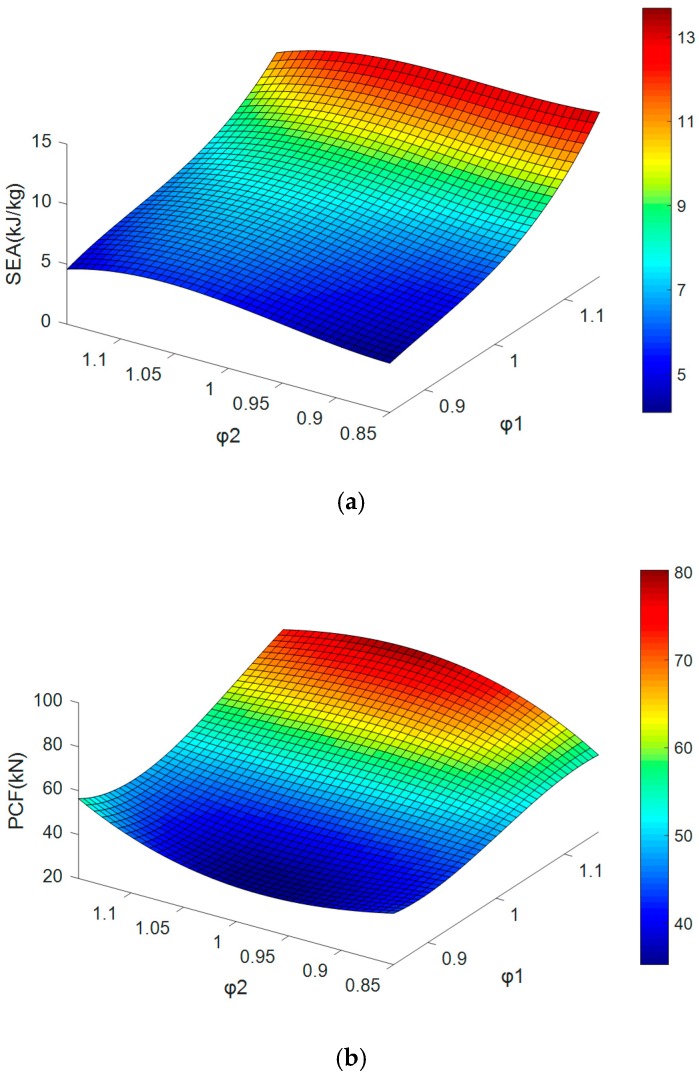
(**a**) The relationship between SEA and two coefficients, and (**b**) the relationship between PCF and two coefficients.

The simulation results of SEA and PCF are fitted by a cubic surface as illustrated in Figure 12. Generally, the bigger *φ*_1_ and *φ*_2_, the bigger SEA and PCF. The slopes are steeper along the *φ*_1_ axis. Accordingly, it can be summarized that the wall thickness change between 0th-order and 1st-order has a bigger effect on the SEA and PCF.

## 6. Conclusions

A bioinspired design strategy has been proposed in the present work. Based on that, a novel fractal thin-walled triangle column (FTTC) has been designed. It is investigated up to the second-order and shows a great improvement in the crash indicators. A parameter study has been conducted to study the influence of geometric details. The conclusions are summed up as follows:

(1) A bioinspired design strategy is proposed and shows great potential to advance the crashworthiness of the thin-walled column. The fractal order has a major influence on performance. In particular, SEA of the 2nd-order FTTC is 89.6% higher than that of the 0th-order.

(2) The theoretical models have been deduced to predict the average crush force of FTTC, and it shows a good agreement with the FEA model.

(3) The different collapse mode of the FTTC has been studied. It finds that the fractal order has a significant influence on the collapse mode. With the increase of *t* and *D*_0_, it shows a tendency to transfer from the unstable to stable, whereas the change of parameter *H_e_* shows the opposite trend.

(4) Changing the fractal thickness of different orders has an effect on crashworthiness by rearranging the material distribution. The wall thickness change between 0th-order and 1st-order has a bigger effect on the SEA and PCF.

## Figures and Tables

**Table 1:** Configuration details of fractal curves.

Order n	Strategies	Section	Side Length	Side Number	Sides Length	Thickness
			D	Q	L	t
0th	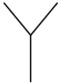	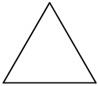	*D*_0_	3	3*D*_0_	*t*_0_
1st	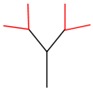	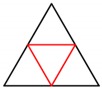	*D*_0_/2	3^2^	3^2^*D*_0_/2	2*t*_0_/3
2nd	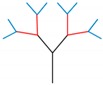	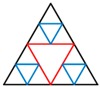	*D*_0_/4	3^3^	3^3^*D*_0_/4	4*t*_0_/9

**Table 2:** Mechanical properties of aluminum alloy AA6061-O.

Properties	Value
Density (kg/m^3^)	2700
Young’s modulus (GPa)	68.9
Poisson’s ratio	0.33
Initial yield stress (MPa)	68.8
Ultimate stress (MPa)	134.2
Power law exponent	0.18
Rupture strain	0.19

**Table 3:** Comparison between experimental test and numerical analysis.

Indicators	Experiment	FEA	Relative Error
EA (J)	2053.9	1793.01	12.7%
SEA (J/kg)	4226.2	3689.3	12.7%
MCF (kN)	14.65	12.83	12.4%

**Table 4:** Configuration details of FTTC series.

Order n	Side Length (mm)	Height (mm)	Thickness (mm)
0	100	300	2.000
1	50	300	1.333
2	25	300	0.889

**Table 5:** Comparison of the FEA and theoretical predictions of the average crush force.

Order n	MCF (kN)	Theoretical (kN)	Relative Error (%)
0th	12.76	12.27	3.84
1st	17.00	18.38	7.51
2nd	24.21	27.73	14.54

**Table 6:** Crashworthiness indicators of FTTC series.

Indicators	0th-Order FTTC	1st-Order FTTC	2nd-Order FTTC
SEA (kJ/kg)	3.94	5.25	7.47
MCF (kN)	12.76	17.00	24.2
CFE (%)	60.42	70.36	71.56
PCF (kN)	21.12	24.16	33.83

## References

[1] Wang Q., Hou R., Li J., Ke Y. (2018). Analytical and experimental study on deformation of thin-walled panel with non-ideal boundary conditions. Int. J. Mech. Sci..

[2] Golewski P., Gajewski J., Sadowski T. (2017). Optimization of a thin-walled element geometry using a system integrating neural networks and finite element method. Arch. Metall. Mater..

[3] Duddeck F., Hunkeler S., Lozano P., Wehrle E., Zeng D. (2016). Topology optimization for crashworthiness of thin-walled structures under axial impact using hybrid cellular automata. Struct. Multidiscip. Optim..

[4] Xu F., Wang C. (2016). Dynamic axial crashing of tailor-welded blanks (TWBs) thin-walled structures with top-hat shaped section. Adv. Eng. Softw..

[5] Zhang S., Pedersen P.T., Ocakli H. (2015). Collisions damage assessment of ships and jack-up rigs. Ships Offshore Struct..

[6] Shekari M.R., Hekmatzadeh A.A., Amiri S.M. (2019). On the nonlinear dynamic analysis of base-isolated three-dimensional rectangular thin-walled steel tanks equipped with vertical baffle. Thin Wall. Struct..

[7] Ma J., You Z. (2014). Energy absorption of thin-walled square tubes with a prefolded origami pattern—Part I: geometry and numerical simulation. J. Appl. Mech..

[8] Geng X., Liu Y., Zheng W., Wang Y., Li M. (2018). Prediction of Crushing Response for Metal Hexagonal Honeycomb under Quasi-Static Loading. Shock Vib..

[9] Alexander J.M. (1960). An approximate analysis of the collapse of thin cylindrical shells under axial loading. Q. J. Mech. Appl. Math..

[10] Abramowicz W. (1983). The effective crushing distance in axially compressed thin-walled metal columns. Int. J. Impact Eng..

[11] Abramowicz W., Wierzbicki T. (1989). Axial crushing of multicorner sheet metal columns. J. Appl. Mech..

[12] Wierzbicki T., Abramowicz W. (1983). On the crushing mechanics of thin-walled structures. J. Appl. Mech..

[13] Chen W., Wierzbicki T. (2001). Relative merits of single-cell, multi-cell and foam-filled thin-walled structures in energy absorption. Thin Wall. Struct..

[14] Zhang X., Zhang H. (2013). Theoretical and numerical investigation on the crush resistance of rhombic and kagome honeycombs. Compos. Struct..

[15] Zhang X., Zhang H. (2012). Numerical and theoretical studies on energy absorption of three-panel angle elements. Int. J. Impact Eng..

[16] Wang S., Peng Y., Wang T., Che Q., Xu P. (2019). Collision performance and multi-objective robust optimization of a combined multi-cell thin-walled structure for high speed train. Thin Wall. Struct..

[17] Tran T., Le D., Baroutaji A. (2019). Theoretical and numerical crush analysis of multi-stage nested aluminium alloy tubular structures under axial impact loading. Eng. Struct..

[18] Zhang X., Zhang H., Yang C., Leng K. (2019). Static and dynamic axial crushing of self-locking multi-cell tubes. Int. J. Impact Eng..

[19] Gui C., Bai J., Zuo W. (2018). Simplified crashworthiness method of automotive frame for conceptual design. Thin Wall. Struct..

[20] Fang J., Sun G., Qiu N., Steven G.P., Li Q. (2017). Topology optimization of multicell tubes under out-of-plane crushing using a modified artificial bee colony algorithm. J. Mech. Design..

[21] Zhang D., Fei Q., Zhang P. (2017). In–plane dynamic crushing behavior and energy absorption of honeycombs with a novel type of multi-cells. Thin Wall. Struct..

[22] Zeng S., Gao Y., Feng Y., Zheng H., Qiu H., Tan J. (2019). Programming the deformation of a temperature-driven bilayer structure in 4D printing. Smart Mater. Struct..

[23] Gao Y., Feng Y., Wang Q., Zheng H., Tan J. (2018). A multi-objective decision making approach for dealing with uncertainty in EOL product recovery. J. Clean. Pod..

[24] Feng Y., Li K., Gao Y., Qiu H., Liu J. (2019). Design and Optimization of Origami-Inspired Orthopyramid-Like Core Panel for Load Damping. Appl. Sci..

[25] Zhang W., Yin S., Yu T.X., Xu J. (2019). Crushing resistance and energy absorption of pomelo peel inspired hierarchical honeycomb. Int. J. Impact Eng..

[26] Xu X., Zhang Y., Wang J., Jiang F., Wang C.H. (2018). Crashworthiness design of novel hierarchical hexagonal columns. Compos. Struct..

[27] Wang J., Zhang Y., He N., Wang C.H. (2018). Crashworthiness behavior of Koch fractal structures. Mater. Des..

[28] Chen T., Zhang Y., Lin J., Lu Y. (2019). Theoretical analysis and crashworthiness optimization of hybrid multi-cell structures. Thin Wall. Struct..

[29] Zhang Y., Xu X., Wang J., Chen T., Wang C.H. (2018). Crushing analysis for novel bio-inspired hierarchical circular structures subjected to axial load. Int. J. Mech. Sci..

[30] Dadrasi A., Beynaghi M., Fooladpanjeh S. (2019). Crashworthiness of Thin-Walled Square Steel Columns Reinforced Based on Fractal Geometries. T. Indian I. Metals..

[31] Zhang Y., Lu M., Wang C.H., Sun G., Li G. (2016). Out-of-plane crashworthiness of bio-inspired self-similar regular hierarchical honeycombs. Compos. Struct..

[32] Ajdari A., Jahromi B.H., Papadopoulos J., Nayeb-Hashemi H., Vaziri A. (2012). Hierarchical honeycombs with tailorable properties. Int. J. Solids Struct..

[33] Neurohr R., Dragomirescu C. Bionics in engineering—Defining new goals in engineering education at “politehnica” University of Coimbra. Proceedings of the International Conference on Engineering Education-ICEE.

[34] Barnsley M.F. (1988). Fractals Everywhere.

[35] Sun G., Pang T., Fang J., Li G., Li Q. (2017). Parameterization of criss-cross configurations for multiobjective crashworthiness optimization. Int. J. Mech. Sci..

[36] Zhang Y., Xu X., Liu S., Chen T., Hu Z. (2018). Crashworthiness design for bi-graded composite circular structures. Constr. Build. Mater..

[37] Zahran M.S., Xue P., Esa M.S., Abdelwahab M.M. (2018). A novel tailor-made technique for enhancing the crashworthiness by multi-stage tubular square tubes. Thin Wall. Struct..

[38] Najafi A., Rais-Rohani M. (2011). Mechanics of axial plastic collapse in multi-cell, multi-corner crush tubes. Thin Wall. Struct..

[39] Tran T., Hou S., Han X., Tan W., Nguyen N. (2014). Theoretical prediction and crashworthiness optimization of multi-cell triangular tubes. Thin Wall. Struct..

[40] Kenyon D., Shu Y., Fan X., Reddy S., Dong G., Lew A.J. (2018). Parametric design of multi-cell thin-walled structures for improved crashworthiness with stable progressive buckling mode. Thin Wall. Struct..

[41] Tran T., Baroutaji A. (2018). Crashworthiness optimal design of multi-cell triangular tubes under axial and oblique impact loading. Eng. Fail. Anal..

